# Supplementary material for: Outcomes and Complications from a Randomized Controlled Study Comparing Conventional Stent Placement Versus No Stent Placement after Ureteroscopy for Distal Ureteric Calculus < 1 cm
Source: J Clin Med. 2022 Nov 28;11(23):7023. doi: 10.3390/jcm11237023 (PMC9740435; doi:10.3390/jcm11237023)
Supplement: Supplementary file 1 [file jcm-11-07023-s001.zip › jcm-2045752-supplementary.pdf]

Supplementary images and tables:

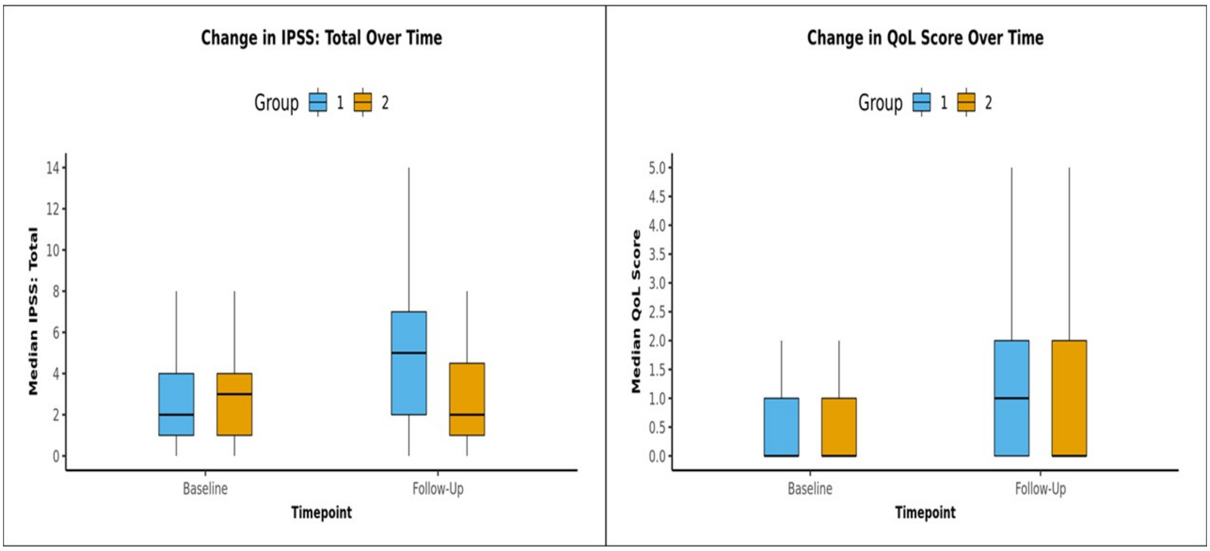

Supplementary Figure S1: The Box-and-Whisker plot depicting the change in domains of IPSS (Total score, QoL) over time in the two groups.

Supplementary Table S1: Summary of Basic Details

Basic Details	Mean ± SD || Median (IQR) || Min-Max || Frequency (%)
**Group**	
CSG	49 (53.3%)
NSG	43 (46.7%)
**Age (Years)**	41.54 ± 11.52 || 41.00 (34.00-49.00) || 19.00 - 69.00
**Age**	
18-30 Years	19 (20.7%)
31-40 Years	25 (27.2%)
41-50 Years	30 (32.6%)
51-60 Years	13 (14.1%)
61-70 Years	5 (5.4%)
**Gender**	
Male	69 (75.0%)
Female	23 (25.0%)
**Stone Location**	
Distal	71 (77.2%)
VUJ	21 (22.8%)
**Stone Size (mm)**	7.53 ± 1.97 || 8.00 (6.00-9.00) || 3.00 - 10.00
**Laterality**	
Left	58 (63.0%)
Right	34 (37.0%)
**Post-Ureteroscopic Lesion Scale**	
Score 0	30 (32.6%)
Score 1	60 (65.2%)
Score 2	2 (2.2%)
**Stent Size**	
5 Fr	17 (34.7%)
6 Fr	32 (65.3%)
